# Piwil2-transfected human fibroblasts are cancer stem cell-like and genetically unstable

**DOI:** 10.18632/oncotarget.14696

**Published:** 2017-01-17

**Authors:** Deying Zhang, Xin Wu, Xing Liu, Chunhong Cai, Guangping Zeng, Jan Rohozinski, Yuanyuan Zhang, Guanghui Wei, Dawei He

**Affiliations:** ^1^ Department of Urology, Children's Hospital of Chongqing Medical University, Ministry of Education Key Laboratory of Child Development and Disorders, Key Laboratory of Pediatrics in Chongqing, Chongqing International Science and Technology Cooperation Center for Child Development and Disorders, Chongqing 400014, China; ^2^ Wake Forest Institute for Regenerative Medicine, Wake Forest University School of Medicine, Medical Center Blvd, Winston-Salem, North Carolina 27103, USA; ^3^ Department of Obstetrics and Gynecology, Baylor College of Medicine, Houston, Texas 77030, USA

**Keywords:** piwil2, fibroblast, gene transfection, cancer stem cell, oncogenesis

## Abstract

Uncontrolled cell proliferation and inhibition of apoptosis are considered to be vital for cancer initiation, maintenance, infiltration, metastasis and recurrence after anti-cancer therapy. Here we report the generation of a novel cell line by reprogramming child foreskin fibroblast with the full length apoptosis inhibitor gene *PIWIL2*. The fibroblasts transfected with *PIWIL2* expressed the stem cell markers *OCT-4, NANOG, SOX-2, KLF-4 and C-MYC*; endoderm marker *AFP* and *GATA6*; mesoderm markers *ACTA2* and *BRACHYURY*; and ectoderm markers *NESTIN and TUBB3*. The karyotype was found to be hyperdiploid. The *PIWIL2* transfected fibroblast cells grew into tumorous masses within 5 weeks of subcutaneous injection into adult nude mice. Although the injected cell expressed markers for all three germlines, ectoderm, mesoderm, and endoderm, they did not form teratomas *in vivo*. This study indicates that the *PIWIL2* gene could play a key role in cancer induction and maintenance. This method for generating induced tumorigenic cells (ITGC) provides a new research tool to study oncogenesis that in turn may lead to a better understanding of cancer etiology and the development of novel anti-cancer therapies.

## INTRODUCTION

Despite the significant progress in our understanding of cancer proliferation and genomics, there has been little progress in understanding cancer origins and early development. Carcinogenesis may involve something as simple as dysregulation and aberrant expression of a single gene, or as complex as the disruption of multiple metabolic and regulatory pathways and a major alteration in the patterns of normal gene expression. No matter what mechanism is involved, carcinogenesis results in the formation of a pool of tumorigenic cells capable of self-renewal, rapid proliferation, differentiation and ability to metastasize from the point of origin. In the clinical setting significant progress made in the treatment of various types of cancer in recent decades. However, even after removal of the primary tumor it is still difficult to entirely eliminate tumorigenic cells from the body. The remaining tumor cells often result in recurrence and metastasis after chemotherapy and/or radiation therapy [1]. The mechanisms underlying malignant tumor regrowth, tissue infiltration, and long-distant metastasis are under intense investigation. Recently a pivotal role for the involvement of tumorigenic cancer stem cells (CSCs) [2, 3] in recurrence and metastasis has been proposed. The CSCs are subpopulations of cells within a tumor that are involved in cancer initiation, maintenance, and propagation [4, 5]. A fundamental property of CSCs is a marked resistance to anti-cancer therapy due to their stem cell like properties, and in particular to their replicative quiescence and the expression of trans-membrane drug transporters [1]. Thus, CSCs can survive therapeutic regimes to repopulate and re-initiate tumors. Only 1/1000-1/5000 of lung cancer, ovarian cancer, and neuroblastoma cells have the stem cell like capacity to form colonies on a soft agar medium and the ability to initiate tumors in *in vivo* animal models [6]. CSCs have the ability to self-renew and to constantly differentiate. Unlike normal stem cells, CSCs display significant genetic heterogeneity and karyotype abnormalities, including chromosome deletion, rearrangement, and duplication [7]. Although the origin and cellular properties of human CSCs are poorly understood the basic characteristics of CSCs, such as enhanced capacity for self-renewal, multipotent differentiation, and tumorigenenity [7–10], are widely accepted. It is therefore highly desirable to be able to generate cell lines *in vitro* that can be used to clarify genetic mechanisms and malignant transformation pathways that lead to tumor development. In turn studying *in vitro* induced tumorigenic cell (ITGC) lines may promote the development of new clinically relevant cancer therapies.

Recent advances in the *in vitro* production of pluripotent stem cells, known as induced pluripotent stem cells (iPSCs), has expanded the field of stem cell biology and opened a potential path for the study of tumorigenesis. IPSCs can be generated from somatic cells, such as fibroblasts, through reprogramming ectopic expression of the transcription factors OCT4 and SOX2, in combination with either Klf4 and C-MYC or LIN28 and NANOG [11, 12]. The iPSCs display many features of embryonic stem cells (ESCs), including a capacity for self-renewal, ability to differentiate into multiple lineages, and form teratomas in *in vitro* animal models [11, 12]. Specifically, transgenic expression of the C-MYC oncogene alters the expression of genes predominantly involved in cellular metabolism, the cell cycle, and protein synthesis pathways [13]. C-MYC expression increases proliferation by down regulating the p53 pathway [14]. This is evidence that modulation of common pathways could be involved in the induction of pluripotency and tumorigenesis [2, 13, 15]. Several recent studies describe attempts to create tumorigenic cells via the reprogramming of ectopic expression with factors like the those used to generate iPSCs [13, 16–18].

P-element induced wimpy testis like 2 (PIWIL2), also known as cancer/testis antigen 80 (CT80), is a small RNA-binding protein that plays a key role in germ cell maintenance in the testis and where its high level of expression does not lead to tumorigenesis. It is a member of the Argonaute family and is widely expressed in colon, breast, prostate, gastrointestinal, ovarian, soft tissue, and endometrial cancers, but not in normal somatic cells and stem cells [17, 19–21]. Piwil2 is a potent inhibitor apoptosis so it may play an important role in tumor induction, proliferation and survival [21]. It has been suggested that PIWIL2 might be a molecular marker of precancerous stem cells and may play an important role in the regulation of tumorigenesis [22–24]. Several peptides originating from alternate mRNA transcripts produced from the PIWIL2 gene have been identified in precancerous stem cells. One of these peptides, Pl2L60, can promote tumorigenesis in the absence of the protein encoded for by the full length PIWIL2 transcript [25]. Recently it has been demonstrated that transfection of mouse embryonic fibroblasts with a full length cDNA copy of the mouse PIWIL2 gene produced cancer stem cell like cell lines *in vitro* [18].

**Figure 1:**
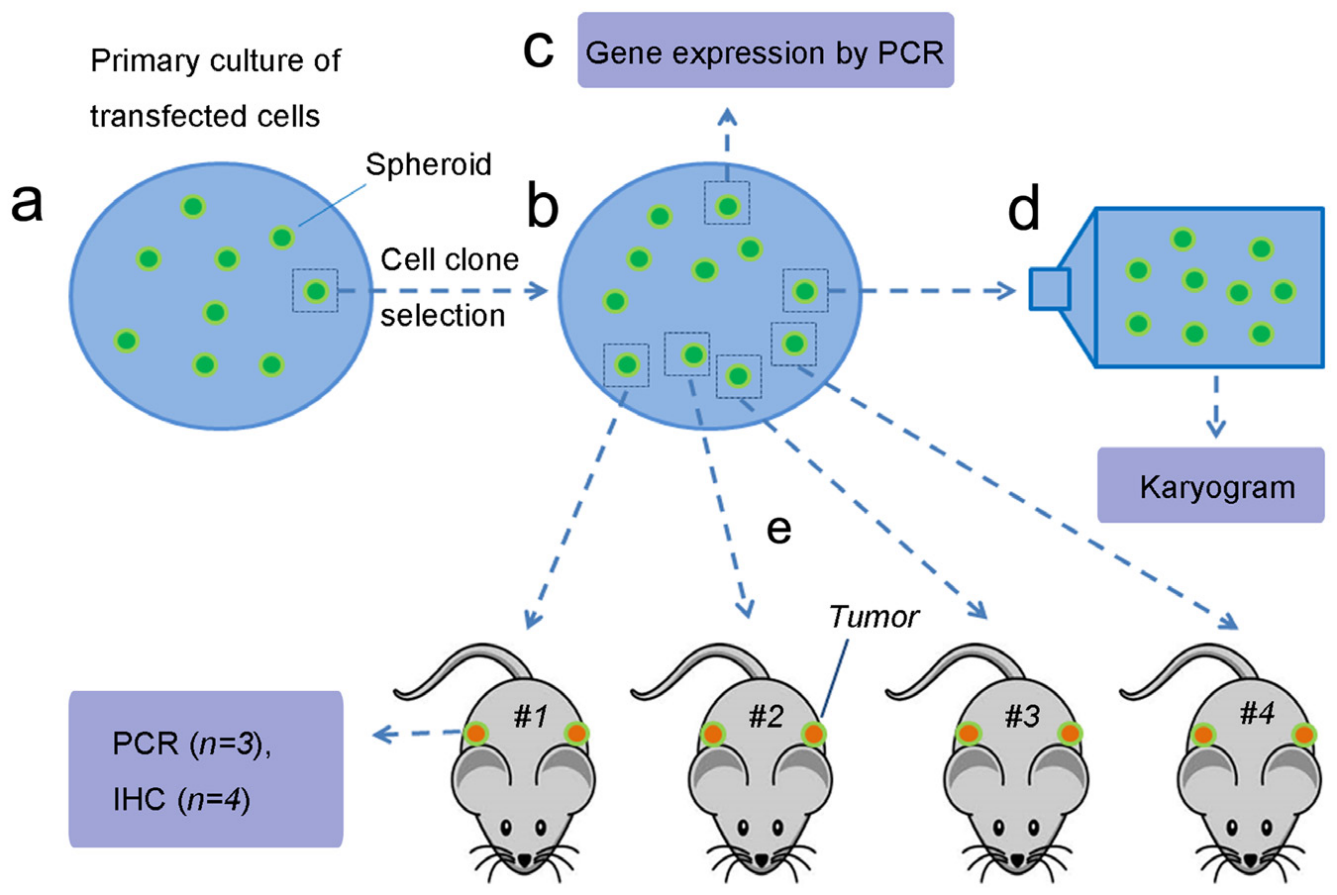
Experimental flow **a**. Human foreskin fibroblasts were transfected with lentivirus containing PIWIL2 and GFP, plated on media containing pluromycin plus LIF and cultured for four weeks until the culture was confluent and spheroids developed. **b**. A single spheroid was harvested, treated with trypsin, and the resultant cells were re-plated. Spheroids that developed after incubation for fourteen days were harvested for further analysis. **c**. Several spheroids were used for gene expression analysis. **d**. A single spheroid was used for karyotyping. **e**. Cells isolated from four spheroids were used for subcutaneous injection into athymic mice (two sites per mouse) for *in vivo* tumor development. Figure was drawn by Deying Zhang.

In this study, we transfected human fibroblasts with a full length coding transcript of the human PIWIL2 gene (Figure 1). The transfected fibroblast displayed many characteristics of typical tumor precursor cells, including self-renewal, clonogenicity, pluripotency, genetic heterogeneity, and ability to initiate highly aggressive pluripotent tumors *in vivo*. We have termed these cells ITGCs. This new experimental model, based on defined reprogramming with *PIWIL2*, provides a potential tool for the *in vitro* study of human tumor initiation and development.

## RESULTS

### Generation and characterization of PIWIL2 transfected fibroblasts

**Figure 2:**
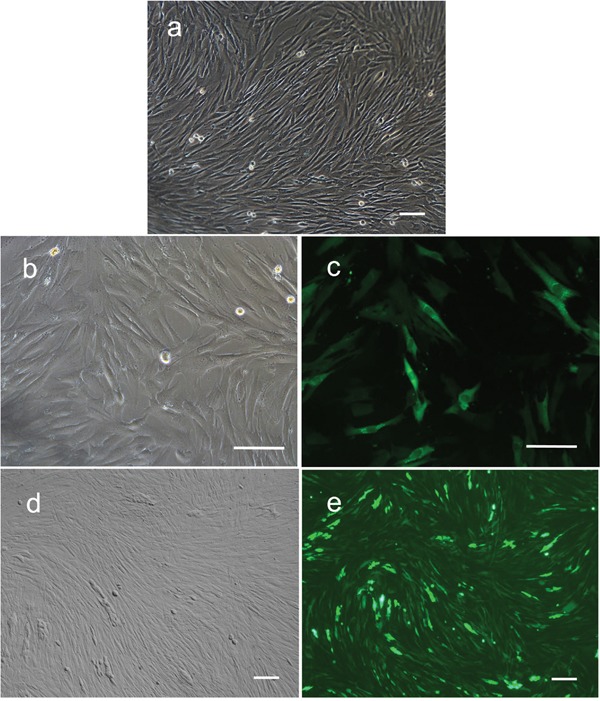
Expression of GFP in human foreskin fibroblasts after transfection with lentivirus containing GFP alone or GFP in combination with PIWIL2 **a**. Non-transfected fibroblasts had typical long spindle shape morphology. **b**. Cells transfected with GFP alone maintained a fibroblast phenotype after infection. **c**. Approximately 50% of these cells expressed GFP. **d**. Cells transfected with GFP in combination with PIWIL2 displayed a predominantly spindle phenotype with patches of small round cells with **e**. 50 to 60% of the cells expression GFP. Panels a, b and d - phase contrast microscopy. Panels c and e - fluorescence microscopy. Bar = 100μm.

**Figure 3:**
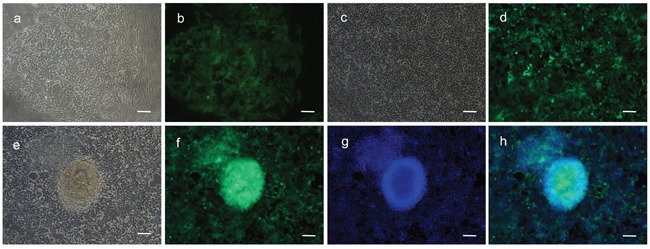
Spheroids formed from fibroblasts transfected with a combination of GFP and PIWIL2 **a**. Two weeks post transfection with a PIWIL2 and GFP combination clusters of small cells **b**. expressing GFP were observed within a background of spindle shaped cells. These clusters expanded in size. **c**. After three weeks of culture the small spheroids were observed within the clusters of small cells and **d**. these spheroids displayed a high level of GFP expression. **e**. At the end of four weeks the spheroids were clearly identifiable. **f**. The spheroids were positive for GFP expression and were formed from densely stacked cells **g, h**. as visualized by Hoechst staining of nuclear DNA. Panels a, c and e - phase contrast microscopy. Panels b, d and f. - GFP fluorescence staining. Panel g - Hoechst fluorescence staining. Panel h- a merged image of f and g showing the overlap between GFP fluorescence and nuclear DNA fluorescence. Bar = 100μm.

**Figure 4:**
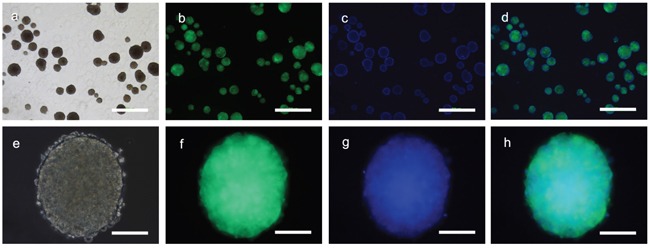
Sub-cultured spheroids Cells derived from a single primary spheroid developed directly into secondary spheroids without a monolayer in the culture dishes. **a**. Multiple spheroids were observed three days after subculture which **b**. expressed GFP and **c**. consisted of a three dimensional cell masses as seen via staining of the cell nuclei with Hoechst dye. **e**. The individual spheroids consisted of small cells that **f**. were GFP positive with **g, h**. a dense central mass. Panels a and e – phase contrast microscopy. Panels b and f – GFP fluorescence microscopy. Panels c and g – Hoechst fluorescence staining. Panels d and h – merged images. Bars panels a to d = 500μm; panels e to h 100μm.

Fibroblasts isolated from child foreskin (see Methods) had typical human fibroblast cell morphology of a long spindle shape (Figure 2a). PIWIL2-GFP transfected fibroblasts and GFP transfected fibroblasts began to show green fluoresce 48 hours after transfection. Transfection efficiency of both cell lines was nearly 50% (Figure 2b-2e). Two weeks after transfection, the morphology of the PIWIL2-GFP transfected fibroblasts expressing GFP gradually changed from a typical long spindle shape to a small spherical shape (Figure 3a, 3b). By 3 weeks post-transfection all the PIWIL2-GFP transfected fibroblasts assumed the spherical morphology (Figure 3c, 3d), and among these cells spheroid like colonies appeared (Figure 3e-3h). One of these spheroid-like colonies was picked for subculture after dissociation by protease treatment. Spheroids reformed 3 days after replating (Figure 4). The GFP transfected fibroblasts and normal fibroblasts showed no significant morphological changes.

### Gene expression profile of PIWIL2 transfected fibroblast spheroids

**Figure 5:**
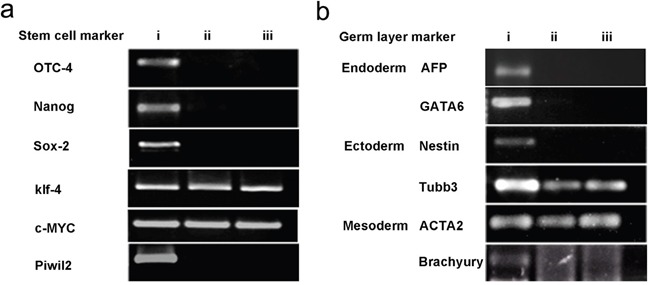
Spheroid generated from GFP-PIWIL2 transfected fibroblasts expressed stem cell markers and markers from all germ layers **a**. Spheroids generated from GFP-PIWIL2 transfected fibroblasts expressed the stem cell markers OCT4, NANOG, SOX2, KLF4, C-MYC and the PIWIL2 transgene (lane i). GFP transfected fibroblasts (lane ii) and un-transfected fibroblasts (lane iii) only expressed KLF4 and C-MYC. **b**. Spheroids generated from GFP-PIWIL2 transfected fibroblasts (lane i) expressed marker genes for all three germ layers, ectoderm, mesoderm and ectoderm. GFP transfected cells (lane ii) and un-transfected cells (lane iii) expressed the ectoderm marker TUBB3 and mesoderm marker ACTA2.

To determine if the PIWIL2 transfected fibroblasts expressed stem cell markers, we tested for the expression of *OCT-4, NANOG, SOX-2, KLF-4, and C-MYC* by RT-PCR. In addition we tested for the expression of germline specific markers; these being the endoderm markers *AFP and GATA6*, mesoderm markers *ACTA2 and BRACHYURY*, and ectoderm markers *NESTIN and TUBB3* (see Methods). The gene expression profiling revealed that the PIWIL2-GFP fibroblast spheroids were positive for expression of the five stem cell markers (*POU5FI, NANOG, SOX-2, KLF-4, and C-MYC*) tested (Figure 5a). They also expressed the endoderm specific marker *GATA6*; mesoderm specific marker, *ACTA2*; and ectoderm specific markers *TUBB3 and NESTIN* (Figure 5b). However, they did not express the mesoderm marker *BRACHYURY*. Both *KLF-4* and *C-MYC* were expressed in GFP transfected-fibroblasts and control fibroblasts. The PIWIL2-GFP transfected spheroids were shown to over expressed *PIWIL2*, compared with GFP transfected cells and un-transfected cells. This fully confirmed that these cell lines were successfully transfected with the *PIWIL2* gene, which was stably inherited and expressed through *in vitro* passage (Figure 5a). The gene expression profile could maintain stable at least up to 40 passages.

### Karyotyping

**Figure 6:**
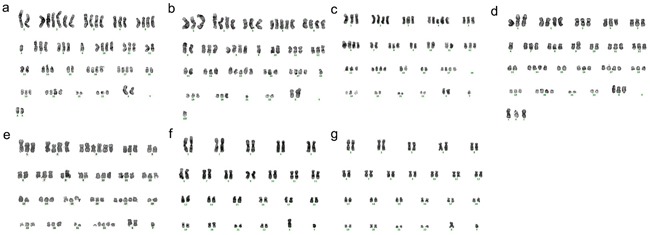
Cells from a spheroid obtained by transfection with PIWIL2 chromosomal instability Individual cells obtained from a single spheroid displayed genetic instability evidenced by a variety of abnormal hyperdiploid karyograms (**a**. through **e**. **a**:70, **b**:67, **c**:67, **d**:68, **e**:70) whereas non-transfected fibroblasts **f**. and fibroblasts transfected with GFP alone **g**. possessed a normal karyotype.

The karyotype of all the PIWIL2 transfected fibroblast cells analyzed was found to be variable and hyperdiploid (Figure 6) indicating genetic instability, whereas that of the GFP transfected fibroblasts and non-transfected control fibroblasts was normal.

### Tumorgenicity of PIWIL2 transfected fibroblasts

**Figure 7:**
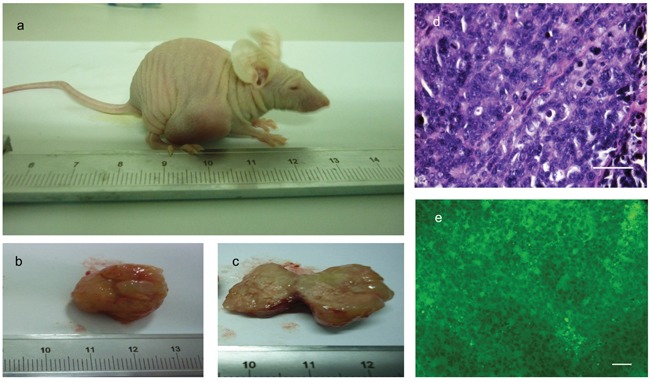
Cells isolated from PIWIL2 expressing spheroids form tumors in athymic mice **a**. Five weeks after subcutaneous injection of cells derived from spheroids tumors were clearly visible at the injection sites. **b**. Excised tumors consisted of a relatively undifferentiated mass of parenchyma which in cross section **c**. showed the presence of grey to pink neoplastic tissue with local hemorrhagic necrosis. **d**. Hematoxylin and eosin staining of thin sections revealed a mass of rather uniform undifferentiated cells with nuclear pleomorphisem, enlarged nuclei, and high nuclear to cytoplasmic ratio. **e**. These malignant cells were positive for GFP expression as seen by fluorescence microscopy indicating that the tumors originated form the human foreskin fibroblasts originally transfected with PIWIL2 plus GFP that were the source of the secondary spheroids used to obtain the cells that were injected into the mice. There is no evidence of teratoma formation. Bar d = 50μm; e = 100μm.

To determine the tumorigenicity of the PIWIL2-GFP transfected fibroblast spheroids *in vivo*, a standard nude mouse model based on subcutaneous injection of PIWIL2-GFP fibroblast spheroid cells was used. GFP transfected fibroblasts and non-transfected fibroblasts were injected as controls (see Methods). The cells from all four PIWIL2-GFP spheroids used as a source of cells for *in vivo* injection developed into small palpable nodules 7-10 days after injection (Figure 7a). Five weeks after injection the tumor capsules were removed from each injection site and the tumors formed from all the four spheroids were larger than 4cm^3^ (Figure 7b, 7c). The small cell tumors consisted of solid parenchyma and gray-to-pinkish neoplastic tissue with local hemorrhagic necrosis inside. No hair or cartilage was found within the masses indicating the absence of teratoma (Figure 7d, 7e). No obvious distant metastasis in lungs, bones, abdominal cavities and brain were observed. Hematoxylin-eosin staining showed anaplastic cells with nuclear pleomorphism, enlarged nuclei, a high nuclear cytoplasmic volume ratio, and a low level of differentiation (Figure 7d). All tumors displayed GFP fluorescence indicating that the tumor cells were primarily of PIWIL2-GFP transfected human fibroblast origin (Figure 7e). No tumor formation was found in nude mice injected with either GFP transfected fibroblasts or un-transfected fibroblasts.

### The tumors derived from PIWIL2-GFP transfected fibroblasts expressed stem cell markers and genes from all three germ layers

**Figure 8:**
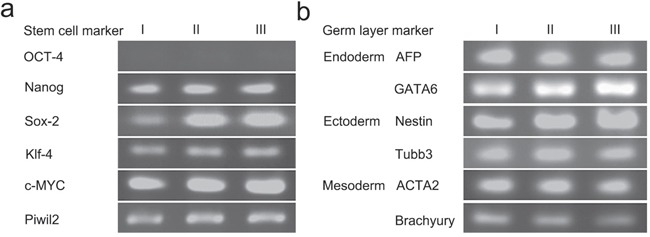
Tumors derived from PIWIL2 expressing spheroids expressed stem cell markers and markers from all germ layers Three tumor masses (lanes I, II and III) were used for gene expression analysis by RT-PCR. **a**. All three tumors expressed the stem cell markers NANOG, SOX2, KLF4, C-MYC and the PIWIL2 transgene but failed to express OCT-4. **b**. In addition, the tumors also expressed the endoderm markers AFP and GATA6; ectoderm markers NESTIN and TUBB3; and mesodermal markers ACTA2 and BRACHYURY.

A single tumor derived from PIWIL2-GFP fibroblast spheroid injected into a nude mouse was used to study gene expression by RT-PCR. The sample expressed stem cell markers (*SOX-2, NANOG, C-MYC, KLF-4*) as well as those from the three germ layers, ectoderm (*NESTIN, TUBB3*); mesoderm (*Brachurury, ACTA-2*); and endoderm (*GATA-6*, *AFP*) (Figure 8). Although *OCT4* was expressed in spheroids, it was not expressed in the tumors suggesting that this gene was silenced *in vivo*.

**Figure 9:**
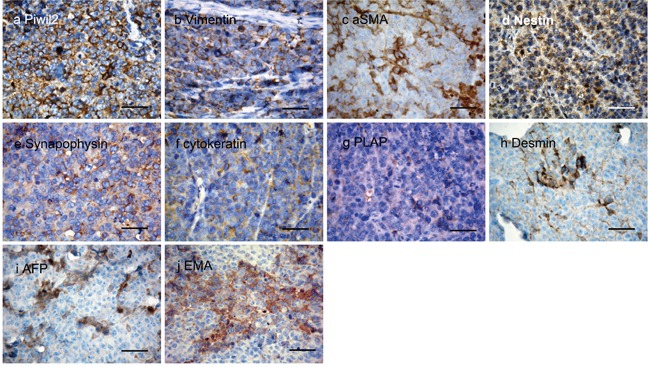
Expression of tissue specific proteins in thin sections of tumor tissue Four tumors were analyzed by immunohistochemistry for the presence of a panel of tissue specific proteins. All four samples were positive for **a**. the transgene encoded peptide PIWIL2 as well as the stromal markers for mesodermal derived tumors **b**. VIMENTIN and **c**. SAM. The neurogenic tumor neurocyte markers **d**. NESTIN and **e**. SYNAPOPHYSIN (SYN) were also detected in all samples. As were **f**. cytokeratin (CK) which marks tumors of epithelial origin, **g**. placental alkaline phosphatase (PLAP) that is a marker for reproductive cell tumors of germ cell origin and **h**. DESMIN, a myocyte marker for mycogenic tumors. In addition tumors expressed **i**. AFP, a fetal cell marker usually present in teratomas and **j**. epithelial membrane antigen (EMA) that marks tumors of epithelial origin. These data indicate that the tumors derived from PIWIL2 transfected human foreskin fibroblasts are poorly differentiated and do not preferentially express any markers associated with mature differentiated somatic cell types. Bar=50μm.

To investigate the tissue type the tumors most closely resembled, immunohistochemistry staining was used to detect tissue specific markers commonly used by pathologists for tumor typing (Figure 9). Four tumors from individual mice were used for immunohistochemical staining. All the PIWIL2-GFP fibroblasts derived tumors tested stained positive for the transfected gene, PIWIL2, as well as VIMENTIN (marker of mesenchymal tumors), Synaptophysin (SYN, markers of neurogenic tumors) and NESTIN (Table 1). Three tumors stained for Placental alkaline phosphatase (PLAP, a marker of reproductive system tumors), cytokeratin (CK, a marker for tumor cells of epithelial origin), desmin (DES, a marker of myogenic origin tumor or tissue), and α-fetoprotein (AFP, marker of teratoma). α-smooth muscle actin (SMA, marker of vasculogenesis) and Epithelial Membrane Antigen (EMA, a marker of epithelial tumor cells) were expressed in two of the tumors. While MYOD1 (children myogenic tumor marker), chromogranin A (CGA, marker of pheochromocytoma), and leukocyte common antigen (LCA, CD45) were not detected in any of the tumors (Table 1). These data indicate that the tumors derived from PIWIL2-GFP transfected fibroblast spheroids do not fall into a clear clinically defined phenotype that can be assigned to a particular tissue type. In addition, gene expression within the various tumors derived from the PIWIL2-GFP transfected fibroblast speroids was variable, indicating that even though they were of clonal origin the patterns of gene expression diverged during *in vivo* tumorigenesis. These tumors are best described as undifferentiated small cell tumors.

**Table 1:** Tissue specific markers expression of piwil2 transfected human fibroblasts originated tumors detected by immunohistochemistry staining

Marker	Tumor Sample I	Tumor Sample II	Tumor Sample III	Tumor Sample IV
PIWIL2	+	+	+	+
VIMENTIN	+	+	+	+
SYN	+	+	+	+
NESTIN	+	+	+	+
PLAP	+	+	-	+
CK	+	-	+	+
DES	-	+	+	+
AFP	-	+	+	+
SMA	-	+	+	-
EMA	-	+	+	-
CGA	-	-	-	-
MYOD1	-	-	-	-
LCA	-	-	-	-

## DISCUSSION

In this study we demonstrate that cells with tumor-genic properties can be generated *in vitro* from human somatic cells by reprogramming with the *PIWIL2* gene. The reprogramming of terminally differentiated cells into tumorigenic cells upon transformation with *PIWIL2* is reminiscent of the dedifferentiation of somatic cells into induced pluripotent stem cells [2] (iPSC) by transfection with the transcription factors OCT4 and SOX2, in combination with either Klf4 and C-MYC or LIN28 and NANOG [11, 12]. Several studies have suggested that the molecular circuitry controlling pluripotency in iPSCs is also important for tumor-initiation and development of cancer cells [26]. In fact, the pluripotent cell markers OCT4, SOX2, Klf4 and NANOG have been shown to be expressed in certain cancer cell types and to play an important role in oncogenesis [2, 27, 28]. It has been suggested that somatic cell reprogramming and transformation into CSC might occur through related pathways [26, 29].

As reviewed by Simpson *et al* [30], many genes that are normally specifically expressed during spermatogenesis, and in male germline stem cells in particular, are also expressed in a wide range of tumor cells. These so called cancer/testis antigens (CT antigens) are active in cancer stem cells, and in the absence of mutations within known oncogenes or tumor-suppressor genes the CT antigens can mediate the malignant phenotype. One of these CT antigens, PIWIL2 (CT80), a member of the P-element-induced wimpy testis/Argonauts (PIWI/AGO) gene subfamily, is of particular interest. Members of this gene subfamily are characterized by conserved PAZ and PIWI domains and were the first class of genes identified to be required for spermatogonial and embryonic stem cell renewal in a diverse group of organisms [21, 23, 31]. In adult tissues PIWIL2 is silenced in both somatic and stem cells, but is widely expressed in most types of cancers [3, 17, 20, 32]. In particular it is stably expressed in primary cancer stem cells (pCSCs), suggesting that it might play an important role in tumor initiation and progression [23, 31]. The potential importance of PIWIL2 in the development of cancer stem cells has recently been demonstrated by the production of cancer stem cell like cell lines *in vitro* by transfection of mouse embryonic fibroblasts with a full length cDNA copy of the mouse PIWIL2 gene [18].

In this paper we report the reprograming of human foreskin fibroblasts, which are of mesenchymal cell lineage, with PIWIL2, and demonstrate that cells with CSC properties can be generated *in vitro*. We used a full-length copy of the PIWIL2 coding sequence carried by a lentivirus vector to transfect human fibroblasts. Transfected cells were cultured on media containing puromycin (selection for transfected cells) and LIF (differentiation suppressor) (see methods). The morphology of PIWIL2-GFP transfected fibroblasts turned from a long spindle shape to a round shape that gradually became smaller with increasing culture time. Once colonies were established spheroids formed in the PIWIL2-GFP transfected fibroblast cultures; a morphological developmental characteristic of ESCs and CSC-like cells [33]. Transformed cells and spheroids were maintained in Dulbecco's modified Eagle's medium (DMEM) supplemented with 10% fetal bovine serum (FBS), which is significantly different from cultural conditions traditionally used to maintain ESCs or iPSCs [6, 11]. Isolated spheroid clones were then assayed for the following stem cell characteristics: capacity self-renew, pluripotency, tumorigenicity, and heterogeneity [8, 10, 13, 34, 35]. Oct-4, SOX-2, and NANOG are known key factors in the cell pluripotent regulatory network and in regulating downstream genes associated with self-renewal, pluripotency, and differentiation [2, 27]. RT-PCR analysis showed that PIWIL2-GFP fibroblast spheroids expressed mRNA from the stem cell associated genes Oct-4, NANOG, and Sox2. Expression of three germ layers gene markers verified the expression of genes associated with all three germ layers and established potential pluripotency. Both Klf4 and C-MYC, which are known to be expressed in normal somatic cells [36, 37], were also found to be expressed in GFP transfected fibroblasts and control fibroblasts. Cells derived from a single spheroid obtained from PIWIL2 gene transfected fibroblasts displayed variable hyperdiploid karyotypes. This demonstrates that PIWIL2 expression promotes genetic instability that is reminiscent of the abnormal chromosomal segregation and aneuploidy that is characteristic of many types of cancer [30].

Cells from four randomly chosen PIWIL2-GFP fibroblast spheroids injected into nude mice developed large tumors at each of eight injection sites, indicating a high level of tumorigenicity. These anaplastic tumors processed cells with a high nuclear to cytoplasmic ratio, had severe nuclear pleomorphism, aberrantly high mitotic rates, and multiple pathological mitotic figures. Immunohistochemical staining of tumor samples showed the expression of markers associated with all three germ layers. The tumors did not form hair, bone or cartilage tissues that are characteristic of teratomas, thus it is concluded that the observed tumors were not teratomas. No individual tumor expressed all markers tested for, indicating that the individual tumors generated from PIWIL2-GFP transfected fibroblasts spheroids display features of distinct differentiation ability and pluripotency, which is consistent with the high level of genetic instability observed in the spheroids. In addition, the lack of consistent expression of markers used for typing tumors according to their site of origin indicates that the process of transforming fibroblasts with PIWIL2 does not result in subsequent differentiation into specific mature cell lineages. Relative low expression of PIWIL2 in iPSCs and ESCs [38] and the inability to form teratoma demonstrates that the PIWIL2-GFP transfected fibroblasts differ significantly from iPSCs and ESCs. The tumors obtained from PIWIL2-GFP transfected fibroblasts spheroids are best categorized as undifferentiated small cell tumors.

In conclusion, our findings demonstrate that human fibroblasts possess enough plasticity to be transfected into tumorigenic cells by transfection with the spermatogonial stem cell self-renewal factor PIWIL2. Transfected PIWIL2-GFP fibroblasts exhibit multiple characteristics associated with cells capable of initiating and maintaining cancers. These include enhanced self-renewal capability, clonogenicity, and perhaps most importantly, the ability to initiate highly aggressive, pluripotent tumors *in vivo*. It is likely that PIWIL2 is a key gene involved in the initiation, development and maintenance of human cancers. Reprogramming of fibroblasts with PIWIL2 into ITGCs affords a novel approach to study the molecular mechanisms involved in tumor initiation, maintenance and differentiation.

The limitation of this study was that lentiviral vector was used to overexpress PIWIL2 in fibroblasts, the neoplastic formation may arise from disruption of a gene, a promoter or a regulatory element due to random insertion sites, expect for PIWIL2 overexpression. Whole genome mapping is needed for fibroblasts transfected with PIWIL2 to determine the insertion site. In future studies a larger number of primary spheroids needed to be used for biological characterization and tumor formation.

## MATERIALS AND METHODS

### Human foreskin fibroblasts collection and culture

Primary foreskin fibroblasts were collected from the circumcised foreskin tissue of a 6-year-old child as described by Rozenchan [39]. Before tissue collection the child's parents signed an informed consent, the experiments were approved by the Medical Ethics Committee in the Children's Hospital, Chongqing Medical University (Permit Number: 2010033), and were carried out in accordance with Declaration of Helsinki. Primary and transfected fibroblasts were maintained at 37°C, 5% CO_2_ in DMEM (Gibco, USA) supplemented with 10% FBS (Hyclone, USA) and Leukemia Inhibitory Factor (LIF, 1000 units/ml). The experimental group was transfected with PIWIL2-GFP, the control groups included non-transfected fibroblasts and fibroblasts transfected with vector containing GFP alone.

### Construction of PIWIL2 recombinant lentiviral vectors

The lentivirus used for fibroblast transfection contain either the pLenO-DCE-GTP transfer vector (GFP control) or the pLenO-DCE-GTP vector containing a full length cDNA copy of the human PIWIL2 gene (PIWIL2-GFP) (NCBI reference sequence ID, NM_001135721), and was constructed by Western Biotechnology Co. (Chongqing, China). PCR and DNA sequencing using the PIWIL2 primers: F; 5 - ATGGATCCTTTCCGACCATCG - 3, R; 5 - TCACAGGAAGAACAGGTTCTC - 3 confirmed the accurate insertion of the PIWIL2 cDNA into the vector. For preparation of the recombinant lentiviral vectors, a four-plasmid mix containing the above vectors plus pRsv-REV, pMDlg-pRRE, pMD2G were cotransfected into 293T cells in serum-free medium using a standard calcium phosphate method. After incubating for 8 hours, the fresh DMEM medium supplemented with 10% FBS and 1% Pen/Strep was completely exchanged. The supernatant was harvested 72 hours later and filtered through a 0.45-μm filter. The supernatant was centrifuged at 65000 *g* for 2h at 4°C, and the resultant pellet was re-suspended with cold PBS. The biological titer (BT) used to transfect foreskin fibroblast was 5.0×10^8^ TU/μl. The PIWIL2 gene was driven by the CMV promoter and the downstream GFP marker by the EF1α promoter.

### Lentiviral vectors mediated transfection of fibroblasts and primary culture

The human fibroblasts were cultured in 24-well plates containing serum-free DMEM medium, and upon reaching 70% confluence were transfected with either 0.5ul recombinant PIWIL2 lentivirus or 0.5ul GFP lentiviral vector (negative control group). Non-transfected cells served as a blank control group. 1% Polybrene (Sigma, USA) was added as an enhancing reagent to improve transfection efficiency. After 8h, the complete medium was changed. The transfected cells were selected on 1μg/ml puromycin, and 1000U/ml LIF was used to suppress differentiation [12]. 4 days post-transfection, the cells formed a confluent layer within which spheroids were observed. These spheroids were harvested and GFP expression was quantitated using fluorescence microscopy (see Figure 1 for experimental design).

### Spheroid sub-cloning and characterization of transfected fibroblasts

A spheroid from the PIWIL2-GFP transfected cells was collected and after treatment with 0.25% trypsin, re-suspended in DMEM containing 10% FBS and transferred onto 15cm plates for secondary spheroid development. Spheroids formed 3 days after re-plating. A group of spheroids was combined and used to study expression of stem cell-associated markers and multiple lineage derivative gene expression by RT-PCR assay. A single spheroid was also seeded into a 25cm^2^ flask, and upon reaching 60%-70% confluence cells were harvested for karyotyping by the Molecular Diagnostic Center in Chongqing Medical University. In addition 4 spheroids were randomly collected to study tumor formation in nude mice.

### Tumor formation

Adult male nude mice were used for an *in vivo* experiment of tumor formation analysis. The experiments were performed in accordance with relevant guidelines and regulations approved by the Ethics Committee in the Children's Hospital, Chongqing Medical University (Permit Number: 2015-21). To test for tumor formation cells were injected subcutaneously into nude mice. Cells isolated from four PIWIL2-GFP fibroblast spheroids were injected into eight sites (two per spheroid), cells from GFP- fibroblast transfected colonies were injected into three sites, and cells from control fibroblast clones were also injected at three sites. Two injection sites per mouse were used and injected with 2×10^6^ cells (1×10^7^ cells/ml in suspension) per site. Mass formation and development at the site of injection was observed every day (see Results). All tumors were resected 5 weeks after injection. The fresh tumor tissues were collected and prepared for RT-PCR, hematoxylin and eosin staining, and immunohistochemistry detection.

### Reverse transcription polymerase chain reaction (RT-PCR)

Total RNA from non-transformed and transformed cells (spheroid and fresh tumor tissues) was extracted using an RNA extraction kit (Bioteke, China) followed by reverse transcription with a reverse transcription kit (Takara, Japan) for RT-PCR. RT-PCR was performed as follows: denaturing for 3 min at 95°C followed by a sequence of denaturing for 30s at 95°C, annealing for 45s at 58-65°C for all primers, extension for 5min at 72°C for 30 cycles (Bioteke, China). PCR products were resolved on a 2% agarose gel. Primers and specific annealing temperatures used in this study are listed in Table 2. RT-PCR analyses of the cDNA samples were done in triplicate.

**Table 2:** Primer sequence for PCR

Gene		Primer sequence	Tm (°C)	Length (bp)
**Stem cell marker**	**C-Myc**	F: CTTCTCTGAAAGGCTCTCR: TGCTGGTAGAAGTTCTCC	62	182
	**OCT-4**	F: GCTCTGCAGCTTAGCTTCAAR: GTTGTGCATAGTCGCTGCTT	59	319
	**Nanog**	F: CTTCTGCTGAGATGCCTCR: GACCGGGACCTTGTCTTC	59	158
	**Klf-4**	F: ACTTTGGGGTTCAGGTGCR: CGAACGTGGAGAAAGATG	60	133
	**Sox-2**	F: GGAGCTTTGCAGGCCGTTTGR: GGAAAGTTGGGATCGAACAA	64.5	460
	**Piwil2**	F: AGCATGAGGTTCGGCATGTTR: ARGGCATGCATGACATCCAG	60	428
**Endoderm**	**AFP**	F: AATGCTTCCAAACAAAGGR: TATGGCTTGGAAAGTTCG	59	116
	**GATA6**	F: GACGTGAGCATGTACCCTAGCR: GCGTAGCCATTCCAGTCCT	61	210
**Ectoderm**	**Nestin**	F: TTGCCTGCTACCCTTGAGACR: GGGCTCTGATCTCTGCATCTAC	61.5	145
	**Tubb3**	F: TCTTCTCACAAGTACGTGCCTR: CCCCACTCTGACCAAAGATGAA	58	127
**Mesoderm**	**ACTA2**	F: CAGGGCTGTTTTCCCATCCATR: ACGTAGCTGTCTTTTTGTCCC	62	81
	**Brachyury**	F: CTATTCTGACAACTCACCTGCATR: ACAGGCTGGGGTACTGACT	60	146
	**GAPDH**	F: GTCAGTGGTGGACCTGACCTR: CACCACCCTGTTGCTGTAGC	61	256

### Histological analysis and immunohistochemistry (IHC) staining

Tumors tissues were fixed in 10% Neutral Buffered Formalin for 24 hours and then processed using a routine paraffin-embedding procedure for histologic examination. 5-micrometer thick sections were stained with hematoxylin and eosin. Immunohistochemistry staining was performed using formalin-fixed paraffin embedded tissue sections and standard procedures. Briefly, 5 micrometer tissue sections were deparaffinized and antigen retrival was performed using microwave exposure at 40°C for 30min in a citrate buffer (pH 6.0). After hydrogen peroxide blocking, a human monoclonal primary antibody was used. The sections were then incubated overnight at 4°C with the following primary antibodies: PIWIL2, VIMENTIN, SYN, NESTIN, PLAP, CK, DES, AFP, SMA, EMA, MYOD1, CGA, and LCA (for information of primary antibodies, see Table 3). The sections were then incubated with the secondary antibody (ZSGB-BIO, China, 1:200). Incubation of sections with phosphate-buffered saline (PBS) served as the negative control. Counter staining was carried out using hematoxylin. The results were judged by the Molecular Diagnostic Center in Children's Hospital, Chongqing Medical University.

**Table 3:** Primary Antibodies for IHC

Primary Antibody	Nation	Company	Working Concentration
PIWIL2	UK	Novocastra	1:800
VIMENTIN	UK	Novocastra	1:800
SYN	UK	Novocastra	1:400
NESTIN	UK	Novocastra	1:800
PLAP	Denmark	Dako	1:200
CK	UK	Novocastra	1:1000
DES	UK	Novocastra	1:800
AFP	China	Gene Tech	1:400
SMA	UK	Novocastra	1:800
EMA	UK	Novocastra	1:600
MYOD1	UK	Novocastra	1:100
CGA	UK	Novocastra	1:400
LCA	Denmark	Dako	Working Solution

## Abbreviations

AFP: α-fetoprotein
CGA: Chromogranin A
CK: Cytokeratin
CSCs: cancer stem cells
DES: Desmin
DMEM: Dulbecco's modified Eagle's medium
EMA: Membrane Antigen
ESCs: embryonic stem cells
FBS: fetal bovine serum
iPSCs: induced pluripotent stem cells
ITGC: induced tumorigenic cells
LCA: Leukocyte Common Antigen
PIWIL2: P-element induced wimpy testis like 2
PLAP: Placental alkaline phosphatase
SMA: smooth muscle actin
SYN: Synaptophysin
